# Psychiatric Axis I Comorbidities among Patients with Gender Dysphoria

**DOI:** 10.1155/2014/971814

**Published:** 2014-08-11

**Authors:** Azadeh Mazaheri Meybodi, Ahmad Hajebi, Atefeh Ghanbari Jolfaei

**Affiliations:** ^1^Shahid Beheshti University of Medical Sciences, Tehran, Iran; ^2^Mental Health Research Center, Faculty of Behavioral Sciences and Mental Health, Tehran Institute of Psychiatry, Iran University of Medical Sciences, Tehran, Iran; ^3^Minimally Invasive Surgery Research Center, Faculty of Behavioral Sciences and Mental Health, Tehran Institute of Psychiatry, Iran University of Medical Sciences, Tehran, Iran

## Abstract

*Objectives*. Cooccurring psychiatric disorders influence the outcome and prognosis of gender dysphoria. The aim of this study is to assess psychiatric comorbidities in a group of patients. *Methods*. Eighty-three patients requesting sex reassignment surgery (SRS) were recruited and assessed through the Persian Structured Clinical Interview for DSM-IV Axis I disorders (SCID-I). *Results*. Fifty-seven (62.7%) patients had at least one psychiatric comorbidity. Major depressive disorder (33.7%), specific phobia (20.5%), and adjustment disorder (15.7%) were the three most prevalent disorders. *Conclusion*. Consistent with most earlier researches, the majority of patients with gender dysphoria had psychiatric Axis I comorbidity.

## 1. Introduction

Gender identity disorder (GID), transsexualism (TS), or gender dysphoria is characterized by subjective experience of one's individuality as belonging to the opposite sex and a feeling of discomfort with one's own biological sex [1, 2]. DSM-IV-TR [3], DSM V [4], and the tenth edition of the ICD-10 include descriptions of gender identity disorder or gender dysphoria in childhood and adulthood. GID is rare worldwide. Lifetime prevalence varies from 0.001%-0.002% [5] to 0.0019%–0.0024% [6]. In previous epidemiological studies, the ratio of female-to-male (FTM) type to male-to-female (MTF) type was 3 to 5 [5]. Furthermore, according to more recent epidemiological studies such as that by de Cuypere et al. (2007), prevalence of MTF and FTM types has been estimated at 1 : 12,900 and 1 : 33,800, respectively, and ratios of MTF to FTM have been reported as 2.43 : 1 [7].

In an Iranian study conducted in a private clinic from 1989 to 1995, 57 GID patients were diagnosed. A total of 37 (64.9%) were MTF, and 20 (35.1%) were FTM [8].

It was shown that cooccurring psychiatric disorders can influence the outcome and prognosis of GID [9, 10]; accordingly, it is valuable to assess psychiatric comorbidities in this group of patients. Numerous clinical and epidemiological researches have been carried out for patients with GID in several countries. However, findings of different studies have shown some inconsistencies. The sex ratio, clinical characteristics, and psychiatric comorbidities reported in these studies were different.

Some studies stated that prevalence of psychiatric disorders in GID patients is similar to controls [11, 12]. On the other hand, some studies demonstrated that GID is associated with higher prevalence of DSM-IV-TR Axis I psychiatric disorders [13, 14]. In addition, several studies have shown that the rate of psychiatric comorbidities was different based on patients' sex and reported that rate of psychiatric comorbidities in FTM was less than that in MTF [15–18].

Research on patients with GID in non-Western countries could be helpful in identifying the similarities and differences of clinical features and comorbidities of GID among nationalities with different cultural, religious, and political orientation influencing attitudes toward sex; however, there is little information about psychiatric characteristics of patients with GID in Iran. According to the scarcity of data in Iran, this study was designed to investigate the prevalence of the DSM-IV Axis I psychiatric disorders comorbidities in Iranian patients. We also compared MTF and FTM patients based on psychiatric comorbidities.

## 2. Method

### 2.1. Subjects

The study was a cross-sectional study conducted at a university affiliated outpatient sex clinic in Tehran Institute of Psychiatry. In Iran, GID patients are referred from the Iranian Legal Medicine Organization to the Tehran Institute of Psychiatry when they request a new official identification or sex reassignment surgery (SRS). Patients who referred to the Tehran Institute of Psychiatry accept treatment with Standards of Care for the Health of Transsexual Protocols before final approval of obtaining a new official identification. Those who met the eligibility criteria in the guidelines of the Standards of Care would receive a final certificate for surgery or obtain a new official identification approved by two experienced psychiatrists.

Between October 2012 and March 2013, 83 patients who met the criteria for GID according to the* Diagnostic and Statistical Manual of Mental Disorders*, 4th edition (DSM-IV-TR), were recruited through convenient sampling. All patients were evaluated through the Persian Structured Clinical Interview for DSM-IV Axis I disorders (SCID-I) by two senior psychiatrists with a special interest in GID management and who were the faculty members of Iran University of Medical Sciences. Four patients were excluded because, in the process of SCID, they did not answer all the questions. The inclusion criteria were as follows: 18 years or older, diagnosis of gender identity disorder according to the DSM-IV TR criteria, and being a native Persian speaker. The presence of mental retardation, neurologic pathology, and chromosomal or hormonal abnormalities were exclusion criteria.

## 3. Instruments

The Persian Structured Clinical Interview for DSM-IV Axis I disorders (SCID-I): SCID is a gold-standard, widely-used clinical tool for diagnosis of psychiatric disorders based on DSM-IV criteria. It is administered by a clinician or trained mental health professional and usually takes from 1 to 2 hours. The Persian version has been shown to have specificity and sensitivity values, reliability, feasibility, and fair to good diagnostic agreement with most diagnostic categories (kappa > 0.6) [19]. SCID was used in this study for the diagnosis of GID and comorbid Axis I psychiatric disorders.

### 3.1. Statistical Analysis

Statistical analysis was conducted using SPSS 17. The chi square test and Fisher's exact test were used as outlined in the text. The significance level was set at *P* < 0.05.

## 4. Results

Seventy-nine patients with GID enrolled in the study. In the group, 43.4% (36) of the patients were FTM and 56.6% (47) were MTF. The mean ± SD age of the MTF and the FTM types was 25.31 ± 7.05 and 25.45 ± 5.4, respectively. Lifetime DSM-IV-TR Axis I psychiatric comorbidities are shown in Table 1. Major depressive disorder (33.7%), specific phobia (20.5%), and adjustment disorder (15.7%) were three most prevalent disorders (see Table 1). Fifty-seven (62.7%) patients had at least one psychiatric comorbidity. Some patients suffered from more than one psychiatric comorbidity (see Table 2). The MTF and FTM types did not differ in having comorbid psychiatric disorders.

## 5. Discussion

In this study, 57 (62.7%) patients had at least one psychiatric comorbidity. Using a similar method to ours (SCID), Haraldsen and Dahl (2000) [11] reported DSM-IV-TR Axis I psychiatric disorders in 33% of 88 GID patients (11). In a study by Bodlund et al. (1993), 10 of 19 GID patients (52.6%) were diagnosed with DSM-IV-TR Axis I psychiatric disorders [14]. After investigating 31 GID patients by clinical interview, Hepp et al. (2005) reported that 12 of the subjects (38.7%) had a current Axis I disorder, while lifetime prevalence of Axis I disorders was 71% [13].

In the Heylens et al. (2014) study, 70% of the patients had lifetime DSM-IV-TR Axis I diagnosis. In addition, 38% of the patients had a current DSM-IV-TR Axis I diagnosis, mostly mood and anxiety disorders [16]. Gómez-Gil et al. (2009) reported lifetime adjustment disorder and social phobias as the most prevalent psychiatric disorders in MTF and FTM patients [17].

Mood disorders and major depressive disorder were the most common Axis I psychiatric disorders (43.4%) in this study. Mehrabi (1996) [8] also reported major depressive disorder as the most common psychiatric comorbidity (35%) in GID patients. Kim et al. (2006) [20] investigated depression in 43 (77%) GID male patients in Korea with the Beck Depression Inventory and based on a cut-off point of 13. In a study conducted by Haraldsen and Dahl (2000), 17.4% of the patients were diagnosed with major depressive disorder (MDD) [11]. Furthermore, a current prevalence (12.9%) and lifetime prevalence of MDD (45.2%) were investigated by Hepp et al. (2005) [13]. In our study, the frequency rate of dysthymic disorder was found to be 7.2%, which is almost near to the value of 4.7% reported by Haraldsen and Dahl (2000) [11] and the value of 6.5% (current prevalence) reported by Hepp et al. (2005) [13].

Many GID patients are reported to suffer from depression, anxiety, negative self-image, low self-esteem, and dissociative symptoms [11, 21, 22]. GID patients suffer from permanent dissatisfaction with several aspects such as gender identity, interpersonal and social relationships, and educational and occupational development. They may find negative attitudes toward themselves from society and their family. Minority stress and social exclusion may predispose them to experience more negative emotions such as anxiety and depression [8, 20, 23, 24]. Attitudes toward GID patients differ from society to society. In countries like Iran and Korea, acceptance by family and society is lower than that in the United States and European countries [8, 20]. The research of Kim et al. (2006) showed that the level of adaptability and cohesion of families of these patients is lower [20], and these may be the causes of relatively high prevalence of depression among these patients. Furthermore, a study by Weinrich et al. (1995) showed that childhood gender nonconformity, a common experience in GID patients, was a predictor of receiving a lifetime diagnosis of depression [25]. In addition, involvement in prostitution is frequent in this group of patients and it may make them prone to experience more traumatic events and subsequently more depression [17].

The prevalence rate of bipolar mood disorder was 2.4% and there were no cases of psychotic disorders. No cases of bipolar mood disorder were reported in the research conducted by Hepp et al. (2005) [13]. The prevalence of psychotic disorders and bipolar disorder was also low in Gómez-Gil et al. (2009) study [17]. However, Haraldsen and Dahl (2000) [11] reported two cases (2.3%) of bipolar mood disorder among 84 patients. Habermeyer et al. (2003) [26] also reported a case of a GID patient in manic episode with psychotic features. Among the 31 patients studied by Hepp et al. (2005), two cases of psychotic disorder NOS (Not Otherwise Specified) existed [13]. Some researchers consider GID in the psychotic spectrum [27], but it does not seem that psychotic disorders are more frequent in patients with GID.

The second most common disorder in Axis I was anxiety disorder, with a 36.1% frequency. Mehrabi (1996) [8] reported anxiety disorders in 19.2% and OCD in 14% of the patients. Haraldsen and Dehl (2000) [11] and Heylens et al. (2014) [16] also marked anxiety disorders as the second most prevalent disorder (18.6%). Moreover, Hepp et al. (2005) [13] discovered current anxiety disorders in 25.8% and lifetime anxiety disorders in 22.6% of their patients. In our research, specific phobia with a 20.5% prevalence rate was the most prevalent anxiety disorder, and OCD and GAD were the next (6.0%). Hepp et al. (2005) [13] found panic disorder as common as specific phobia; however, their frequencies were lower than the findings of our study (6.5% for current and 12.5% for lifetime prevalence). Gómez-Gil et al. (2009) reported social phobia as the most prevalent anxiety disorder [17].

The different prevalence rate of DSM-IV-TR Axis I disorders in GID patients could be the result of different prevalence rates of these disorders in the general population, the number of samples, different sampling, and differences among diagnostic tools.

The frequency of substance abuse was 8.4%, though no substance dependency was observed. In research conducted by Hepp et al. (2005), the current frequency of these disorders was 9.7%, while the life-time frequency was 45.2%, which is significantly higher than our finding [13]. Furthermore, in Haraldson and Dahl (2000) study [11], the prevalence rate of substance abuse was 16.2%, and Cole et al. found a substance-related disorders rate of 25% [28]. In Gómez-Gil et al. (2009) study, alcohol and substance-related disorders were more prevalent in the MTF group [17]. The differences in the abovementioned values can to some extent be correlated with the differences of prevalence of substance abuse and the availability of the substances in different societies; or maybe it is because of patients' avoidance of self-disclosure due to their fear of not having their sexual reassignment approved by the Tehran Institute of Psychiatry.

Several psychiatric disorders were not observed in our study. Psychotic disorders, somatoform disorders, and eating disorders are some examples.

It should be noted that many GID patients do not have any serious current psychiatric problems. However, as Kim et al. (2006) showed, at least some of these patients are referred for GID diagnosis when they face emotional problems [20]. A frequency of 15.7% for adjustment disorder may be related to these cases, which requires more investigation. Some studies reported less psychopathologies in the FTM group than in the MTF group, and they related this finding to a more stable socioeconomic status and better social adjustment of FTM patients. In our sample, there were not any significant differences between the two groups, and we do not have information about their socioeconomic status and social adjustment. Maybe it is because of higher rates of depression and anxiety in the biologically female sex.

The current prevalence of psychiatric disorders was not assessed in our study, and this is one of our research limitations. Employing severity rating scales besides SCID-I would be more appropriate. This study also faced other limitations. We did not evaluate comorbidity with personality disorders in the subjects. Patients in our research might be imperfect samples of all GID patients. In Iran, GID patients are referred to the Tehran Institute of Psychiatry when they request a new official identification or sex reassignment surgery (SRS). Consequently, the sample in our study can be considered a sample of patients whose have more psychiatric problems or more intense conflict with their perceived identity.

## Figures and Tables

**Table 1:** Prevalence rates of lifetime DSM Axis I psychiatric comorbidities in patients.

	Total (83)		Male-to-female		Female-to-male		χ^2^	*P*
	*N*	%	*N*	%	*N*	%		
Major depressive disorder	28	33.7	17	36.2	11	30.6	0.287∗	0.592
Dysthymic disorder	6	7.2	3	6.4	3	8.3	0.116∗	0.734
Minor depressive disorder	5	6	3	6.4	2	5.6	0.025∗∗	1.000
Bipolar I disorder	1	1.2	1	2.1	—	—	0.775∗∗	1.000
Bipolar II disorder	1	1.2	1	2.1	—	—	0.775∗∗	1.000
Mood disorder (total)	36	43.4	23	48.9	13	36.1	1.365∗	0.243

Specific phobia	17	20.5	8	17	9	25.0	0.797∗	0.372
Obsessive-compulsive disorder	5	6	4	8.5	1	2.8	1.183∗∗	0.382
Generalized anxiety disorder	5	6	3	6.4	2	5.6	0.025∗∗	1.000
Social phobia	4	4.8	2	4.3	2	5.6	0.075∗∗	1.000
Panic disorder without agoraphobia	4	4.8	2	4.3	2	5.6	0.075∗∗	1.000
Posttraumatic stress disorder	2	2.4	2	4.3	—	—	1.570∗∗	0.503
Anxiety disorder (total)	30	36.1	18	38.8	12	33.3	0.218∗	0.641

Adjustment disorder	13	15.7	6	12.8	7	19.4	0.688∗	0.407

Alcohol abuse	4	4.8	1	2.1	3	8.3	1.711∗∗	0.312
Nicotine dependence	3	3.6	1	2.1	2	5.6	0.688∗∗	0.576
Sedative, hypnotic, or anxiolytic abuse	2	2.4	1	2.1	1	8	0.037∗∗	1.000
Cannabis abuse	1	1.2	1	2.1	—	—	0.775∗∗	1.000
Amphetamine abuse	1	1.2	1	2.1	—	—	0.775∗∗	1.000
Substance use disorder (total)	7	8.4	3	6.4	4	11.1	0.590∗	0.442

Axis I psychiatric disorder (total)	52	62.7	31	66	21	68.3	0.506∗	0.477

∗Chi square ∗∗Fisher's exact test.

**Table 2:** Number of lifetime DSM Axis I psychiatric comorbidities in patients.

Number of cooccurring psychiatric disorders	Frequency	%
1	23	27.7
2	14	16.9
3	11	13.3
4	2	2.4
5	2	2.4
